# Overexpression of SKA3 correlates with poor prognosis in female early breast cancer

**DOI:** 10.7717/peerj.12506

**Published:** 2021-12-13

**Authors:** Yue Zhong, Zhenjie Zhuang, Peiju Mo, Mandi Lin, Jiaqian Gong, Jiarong Huang, Haiyan Mo, Yuyun Lu, Mei Huang

**Affiliations:** 1 Guangzhou University of Chinese Medicine, Guangzhou, China; 2 College of Basic Medicine, Guangzhou University of Chinese Medicine, Guangzhou, China; 3 Galactophore Department, The First Affiliated Hospital of Guangzhou University of Chinese Medicine, Guangzhou, China

**Keywords:** Breast cancer, SKA3, RT-qPCR, ELISA, TCGA, Prognosis

## Abstract

**Background:**

Spindle and kinetochore associated complex subunit 3 (SKA3) plays an important role in tumorigenesis and the progression of various tumors. But the relationship between SKA3 and early breast cancer remains unclear. The study aimed to explore the prognostic significance of SKA3 in breast cancer.

**Methods:**

In the study, SKA3 expression was initially assessed using the Oncomine database and The Cancer Genome Atlas database (TCGA). Then, we presented validation results for RT-qPCR (quantitative reverse transcription PCR) and ELISA (enzyme-linked immunosorbent assay). The relationship between clinical characteristics and SKA3 expression was assessed by Chi-square test and Fisher’s exact test. Kaplan–Meier method and Cox regression analysis were conducted to evaluate the prognostic value of SKA3. Gene set enrichment analysis (GSEA) was performed to screen biological pathways using the TCGA dataset. Besides, single sample gene set enrichment analysis (ssGSEA) was utilized to identify immune infiltration cells about SKA3.

**Results:**

SKA3 mRNA was expressed at high levels in breast cancer tissues compared with normal tissues. Chi-square test and Fisher’s exact test showed SKA3 expression was related to age, tumor (T) classification, node (N) classification, tumor-node-metastasis (TNM) stage, estrogen receptor (ER), progesterone receptor (PR), molecular subtype, and race. RT-qPCR results showed that SKA3 expression was overexpressed in ER, PR status, and molecular subtype in Chinese people. Kaplan–Meier curves implicated that high SKA3 expression was related to a poor prognosis in female early breast cancer patients. Cox regression models showed that high SKA3 expression could be used as an independent risk factor for female early breast cancer. Four signaling pathways were enriched in the high SKA3 expression group, including mTORC1 signaling pathway, MYC targets v1, mitotic spindle, estrogen response early. Besides, the SKA3 expression level was associate with infiltrating levels of activated CD4 T cells and eosinophils in breast cancer.

**Conclusion:**

High SKA3 expression correlates with poor prognosis and immune infiltrates in breast cancer. SKA3 may become a biomarker for the prognosis of breast cancer.

## Introduction

Among women, breast cancer is the most common malignancy and the main cause of cancer death (Bray et al., 2018). The incidence of breast cancer has increased every year (Kasiappan & Rajarajan, 2017). About 3.8 million women in the United States have been diagnosed with breast cancer, including 268,600 new cases in 2019 (Miller et al., 2019).

Spindle and kinetochore associated complex subunit 3 (SKA3), aliased as RAMA1 and C13orf, is a protein-encoding gene located in the chromosome region 13q12.11. SKA3, a component of the complex related to the spindle and mitochondria, is necessary for normal chromosome separation and cell division. It can mediate kinetochore-microtubule interaction (Raaijmakers et al., 2009) and plays a critical role in the migration of meiotic spindles, the stability of the late spindles, and the accurate timing of the late mitosis (Zhang et al., 2012). Also, SKA3 is involved in the regulation of mitosis and cell proliferation, and apoptosis of the NDC80 complex (Zhang et al., 2017). Previous studies have reported that SKA3, as an essential member of the SKA family, plays a significant in cellular processes such as cell proliferation, invasiveness, migration, and tumorigenesis. Overexpression of SKA3 has been detected in a variety of tumor types including lung (Dan-Dan et al., 2020), rectal (Chu et al., 2016), cervical (Hu et al., 2018), and liver cancer (Hou et al., 2019). SKA3 also serves as a prognostic marker and potential therapeutic target for hepatocellular carcinoma (Jiao et al., 2013). The prognostic value of SKA3 is also identified in renal cell carcinoma (Yamada et al., 2018), rectal adenoma, and bladder cancer (Chuang et al., 2016; Lee et al., 2015). Besides, SKA3 promotes lung adenocarcinoma and cervical cancer metastasis by regulating the cell cycle and the PI3K/Akt pathway (Hu et al., 2020; Hu et al., 2018). Previous studies reported that SKA3 frequently mutates in breast cancer and might be a potential oncogene of breast cancer (Jiao et al., 2013). Tang et al. (2019) reported that SKA3 might be a hub gene to regulate breast cancer metastasis to the brain. A recent study showed that circular RNAcircSKA3 was highly expressed in breast cancer cells and tissues. The combination of circular RNA circSKA3 with integrin β1 could enhance the invasion ability of breast cancer (Du et al., 2020). These findings strongly suggested that SKA3 might be an oncogene and contribute to tumorigenesis. At present, there has been no report on SKA3 in predicting the prognosis of breast cancer. Our study aimed to investigate the importance of SKA3 in breast cancer.

## Materials and Methods

### Data mining and collection

#### Patients

Tumor tissues and paired adjacent tissues were obtained from breast cancer patients in The First Affiliated Hospital of Guangzhou University of Chinese Medicine between 2020 and 2011. These patients were not treated before surgery. All breast cancer patients were in between stage I and III based on TNM staging system. Specimens from surgery were stored at –80 °C. The study was approved by the Research Ethics Committee of The First Affiliated Hospital of Guangzhou University of Chinese Medicine, and patients signed informed consent before operation. IRB number: JY [2020] 098.

#### Oncomine

The Oncomine database (Rhodes et al., 2004) (http://www.oncomine.org), a gene chip-based database and data mining platform was applied for analyzing the expression status of SKA3 in various malignant tumors. In the study, mRNA levels of cancer *vs*. normal patient were compared in the datasets. *P*-value = 1E−4, two-fold change, and top 10% gene rank were selected as the threshold (Rhodes et al., 2004). All statistical methods and results were obtained from Oncomine.

#### The Cancer Genome Atlas

The Cancer Genome Atlas (TCGA) (https://www.cancer.gov/tcga) is a landmark cancer genomics program, which comprises the data of over 20,000 primary cancer and matched normal samples spanning 33 cancer types. The database also provides a large number of samples with a variety of clinical characteristics. The mRNA Seq data and clinical features of breast cancer were extracted from TCGA in June 2020. All tumor samples came from before treatment. Stage I–III and female breast cancer patients were enrolled in the study. After excluding patients without TNM stage, SKA3 expression and intact survival data, 1,040 cases were finally included. Raw count data-normalization and the identification of the differential expression genes between the tumor tissues and normal (adjacent) tissues were analyzed by the package edgeR (Robinson, McCarthy & Smyth, 2010) of R software. The set filter conditions set were as follows: adjusted *P*-value < 0.05 and |log2 (fold change)| > 1.

#### The verification of SKA3 by RT-qPCR

Total RNA was collected from 66 pairs of breast cancer (BC) tissues and adjacent nontumor tissues frozen in liquid nitrogen using TRIzol reagent (Invitrogen, Waltham, MA, USA). Evo M-MLV RT Premix for qPCR (AG11706; ACCURATE BIOTECHNOLOGY, HUNAN, China) was carried out to reverse-transcribe RNA into complementary DNA (cDNA) according to the instruction. SYBR Green Premix Pro Taq HS qPCR Kit (ACCURATE BIOTECHNOLOGY (HUNAN) Co., Ltd., AG11701) was used to analyze RT-qPCR. The data were calculated using the 2−ΔΔCt method (Livak & Schmittgen, 2001). β-actin served as an internal standard control for mRNA expression. The primer sequences were as follows:

SKA3 Forward: 5′-TACACGAGCAAGAAGCCATTAAC-3′ and Reverse: 5′-GGATACGATGTACCGCTCAAGT-3′, β-actin, forward: 5′-TGGCACCCAGCACAATGAA-3′ and reverse: 5′-CTAAGTCATAGTCCGCCTAGAAGCA-3′.

#### The verification of SKA3 by ELISA

SKA3 was measured with the Human Spindle and Kinetochore Associated complex subnit3 (SKA3) ELSIA kit (Fankew, Shanghai FANKEL Industrial Co., Ltd. Shanghai, China) according to the instructions. A total of 38 pairs of breast cancer tissues and matched adjacent tissues were selected to prepare homogenate and the OD value was read at 450 nm within 15 minutes after the end of the reaction.

#### Gene enrichment analysis (GSEA)

Gene set enrichment analysis (GSEA) was performed to determine the biological processes between the low and high groups of SKA3 expression using GSEA software (version 4.0) (Subramanian et al., 2005). We divided the tumour samples into two groups according to the median value of SKA3 expression level and selected the gene set of “h.all.v7.3.symbols.gmt”, downloaded from the the Molecular Signatures Database as the reference gene sets for GSEA analysis. The gene set with |NES| (Normalized Enrichment Score) > 1, FDR (false discovery rate) < 0.25 and normal *P*-value < 0.05 was the significantly enriched gene set (Subramanian et al., 2005).

#### Protein-protein interaction (PPI) network

To construct PPI network about SKA3, a SKA3-associated PPI network was established based on the Search Tool for the Retrieval of Interacting Genes/Proteins database (STRING) (https://string-db.org/) (Szklarczyk et al., 2017) with a minimum required interaction score of >0.9. Then PPI network was analyzed and visualized by Cytoscape v3.7.2 (Shannon et al., 2003).

#### Immune infiltration analysis of SKA3

The transcripts per million (TPM) normalized RNA-seq data of breast cancer were downloaded from TCGA. Twenty-eight immune gene sets, including different classic immune cell types, were obtained from Charoentong et al. (2017). Single-sample GSEA (ssGSEA) was analyzed using R package GSVA (Hanzelmann, Castelo & Guinney, 2013) to assess tumour sample’s immune infiltration level and calculate the responding immune infiltration score in breast cancer. Subsequently, we performed Spearman’s correlation analysis between the SKA3 expression data and the immune infiltration score of 28 immune gene sets. The threshold was set as *P*-value < 0.05 and r ≥ 0.3 to filter the immune cell types with significantly moderate infiltrating level correlation with the SKA3 expression.

#### Statistical analyses

Data analysis was performed using SPSS software 26.0 (IBM Corporation, Armonk, NY, USA) and R version 3.6.2 (R Development Core Team, 2020). The ggplot2 package (Ginestet, 2011) was used to draw boxplots of clinical features according to SKA3 expression variation. The results of RT-qPCR and ELISA were presented using the GraphPad prism8. The Wilcoxon signed-rank sum test and Kruskal–Wallis test were utilized to measure the differential expression of SKA3 in the subgroup, including age, tumor (T), node (N), metastasis (M) classification, tumor-node-metastasis (TNM) stage, estrogen receptor (ER), progesterone receptor (PR), human epidermal growth factor receptor-2 (HER-2) status, race, and vital status. A Chi-square test was used to analyze the relationship between SKA3 mRNA expression and clinical characteristics. SKA3 mRNA expression level was divided into high-SKA3 expression group and low-SKA3 expression group by the optimal cutpoint value using survminer package (Alboukadel Kassambara, 2020) in R software. Kaplan–Meier curves were applied to analyze using survminer package in R software. Then, the univariate and multivariate Cox analyses were performed to determine the related variables.

## Results

### Increased expression of SKA3 in breast cancer

**Figure 1:**
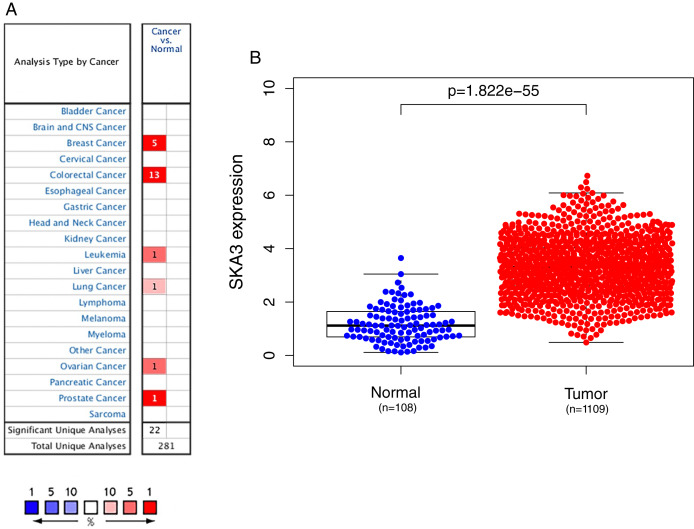
SKA3 expression in different types of cancers. (A) Expression of SKA3 gene in various cancers compared with matched normal tissues by the Oncomine database. Red and blue represent the number of data sets of increasing and decreasing SKA3 gene levels, respectively. The cell color is provided in the best gene rank percentile for the analyses within the cell. (B) Expression of SKA3 in breast cancer tumor tissues and paired adjacent tissues in TCGA database.

We used the Oncomine database to analyze the expression of the SKA3 gene in 20 types of malignant tumors. Elevated levels of SKA3 (red) were observed in breast, rectal, ovarian, bladder, lung, and leukemia (Fig. 1A). Using the TCGA database to analyze the expression of SKA3, it was also found that SKA3 expression in breast cancer tissues was higher than that in normal (adjacent) tissues (Fig. 1B).

### The correlation of SKA3 expression in clinicopathological characteristics of early breast cancer patients

**Figure 2:**
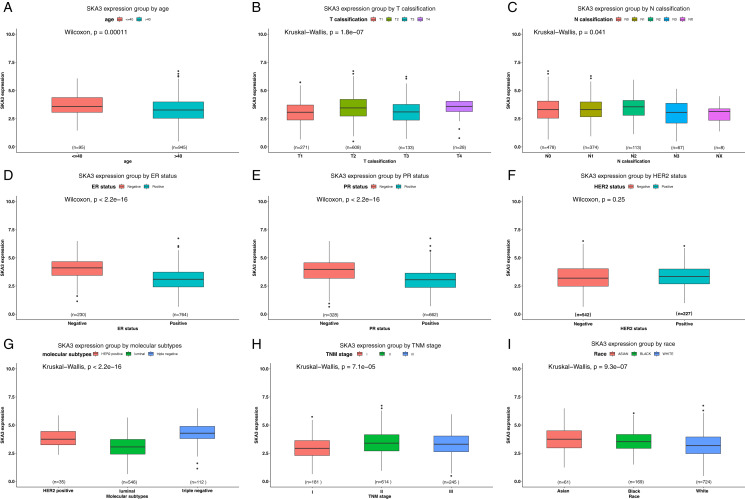
Differential SKA3 expressions in the whole TCGA cohort. The expression of SKA3 is grouped by (A) age, (B) T classification, (C) N classification, (D) ER status, (E) PR status, (F) HER2 status, (G) TNM stage, (H) molecular subtype, (I) and race.

SKA3 expression was divided into high and low expression groups according to the optimal cutpoint value. A Chi-square test was used to analyze the expression of clinical characteristics of SKA3 and breast cancer patients. The results showed that SKA3 expression was significantly associated with age (*P* = 0.021), T classification (*P* < 0.001), TNM stage (*P* < 0.001), ER status (*P* < 0.001), PR status (*P* < 0.001), race (*P* = 0.001), molecular subtype (*P* < 0.001) and vital status (*P* < 0.001) (Table 1). However, there were no significant differences in margin and HER2 status (*P* > 0.05). Similarly, the different SKA3 expression levels between these different clinicopathological groups were further confirmed by analyzing SKA3 expression data as a continuous variable (all *P* < 0.05) (Fig. 2).

**Table 1:** Relationship between SKA3 mRNA expression and clinicopathologic parameters.

Parameters	SKA3-high (*n* = 319)	SKA3-low (*n* = 721)	χ^2^	*P*-value
Age				
≤40	32 (10.03%)	42 (5.83%)	5.301	**0.021**
>40	287 (89.97%)	679 (94.17%)		
T classification				
T1	52 (16.30%)	219 (30.37%)	32.648	**<0.001**
T2	225 (70.53%)	383 (53.12%)		
T3	31 (9.72%)	102 (14.15%)		
T4	11 (3.45%)	17 (2.36%)		
Race^Δ^				
American Indian	0 (0.00%)	1 (0.14%)	16.556	**0.001**
Asian	29 (9.09%)	32 (4.44%)		
Black	62 (19.44%)	107 (14.84%)		
White	197 (61.76%)	527 (73.09%)		
Unknown	31 (9.72%)	54 (7.49%)		
N classification^Δ^				
N0	147 (46.08%)	331 (45.91%)	4.056	0.4
N1	110 (34.48%)	264 (36.62%)		
N2	43 (13.48%)	70 (9.71%)		
N3	17 (5.33%)	50 (6.93%)		
NX	2 (0.63%)	6 (0.83%)		
TNM stage				
I	34 (10.66%)	147 (20.39%)	15.059	**<0.001**
II	208 (65.20%)	406 (56.31%)		
III	77 (24.14%)	168 (23.30%)		
ER				
Negative	140 (43.89)	90 (12.48)	130.32	**<0.001**
Positive	163 (51.10)	601 (83.36)		
Unknown	16 (5.02)	30 (4.16)		
PR				
Negative	176 (55.17)	152 (21.08)	123.93	**<0.001**
Positive	126 (39.50)	536 (74.34)		
Unknown	17 (5.33)	33 (4.58)		
HER2				
Negative	165 (51.72)	377 (52.29)	0.0828	0.960
Positive	69 (21.63)	158 (21.91)		
Unknown	85 (26.65)	186 (25.80)		
Margin				
Close	5 (1.57)	23 (3.19)	5.268	0.153
Negative	274 (85.89)	613 (85.02)		
Positive	17 (5.33)	50 (6.93)		
Unknown	23 (7.21)	35 (4.85)		
Molecular subtype				
Triple negative	80 (25.08)	32 (4.44)	110.54	**<0.001**
HER2 positive	17 (5.33)	18 (2.50)		
Luminal	123 (38.56)	423 (58.67)		
Unknown	99 (31.03)	248 (34.40)		
Vital status				
Alive	264 (82.76)	652 (90.43)	11.673	**<0.001**
Dead	55 (17.24)	69 (9.57)		

**Notes**:

Δ Fisher test.

ER, estrogen receptor; HER-2, human epidermal growth factor receptor-2; PR, progesterone receptor. Bold values indicate statistically significant, *P* < 0.05.

### Overexpression SKA3 predicts poor survival in early breast cancer patients

**Figure 3:**
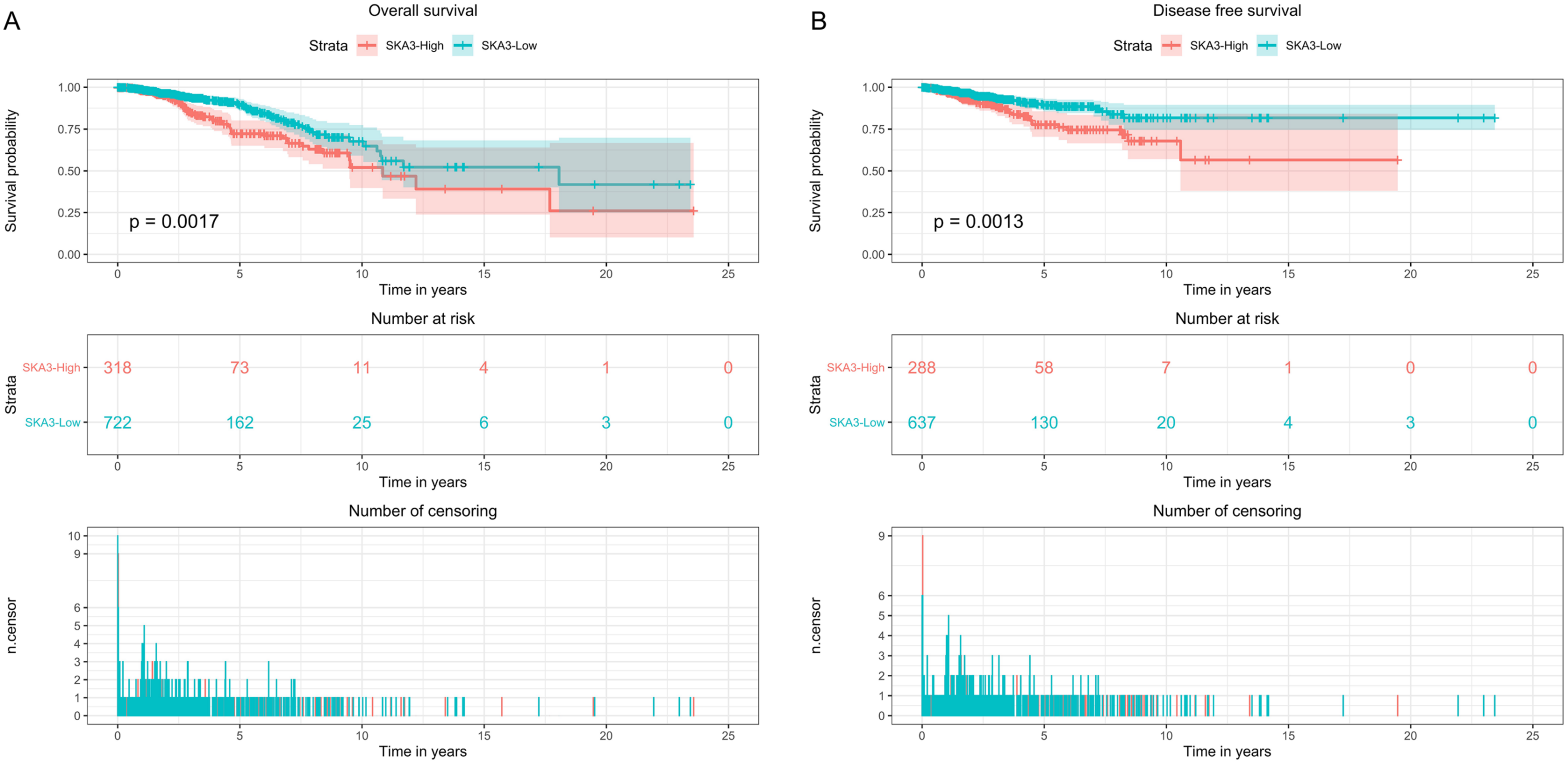
Kaplan–Meier survival analysis curve of SKA3 expression in breast cancer. (A) Overall survival, (B) disease free survival.

Kaplan–Meier curves with the log-rank test were applied for exploring the prognostic value of SKA3 expression using the clinical information downloaded from the TCGA database. The best cutpoint was used to divide gene expression levels into high expression groups and low expression groups by survminer package in R software. The Kaplan–Meier curve showed that high expression of the SKA3 gene was associated with poor overall survival (*p* = 0.0017, Fig. 3A), disease-free survival (*p* = 0.0013, Fig. 3B).

### Univariate and multivariate analyses

**Figure 4:**
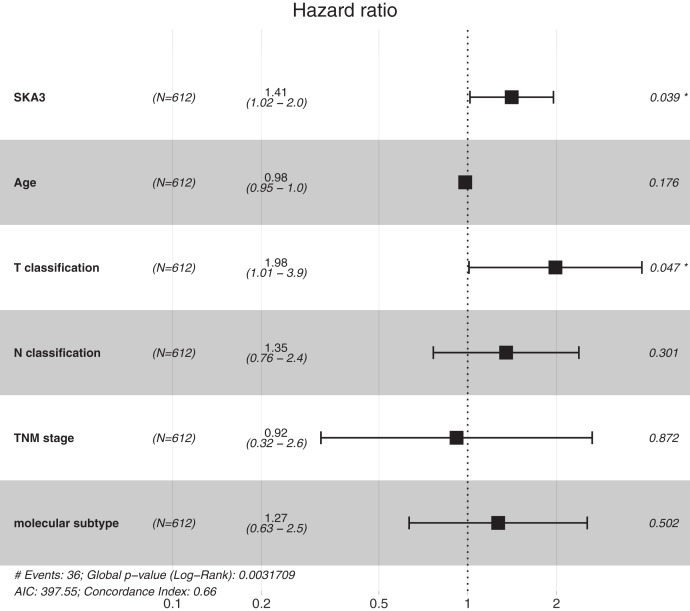
Forest plot for the multivariate Cox proportional hazard regression model. HR, hazard ratio; C.I., confidence interval. *P < 0.05.

After eliminating incomplete clinical data, 612 patients were finally enrolled in the univariate and multivariate analyses. Univariate analysis showed that T classification, N classification, TNM stage and SKA3 were correlated with the prognosis of breast cancer patients. Moreover, the multivariate analysis indicated that high SKA3 expression (HR  = 1.410, 95% C.I. [1.105–2.074], *P* =  0.0039) and T classification (HR = 1.984, 95% C.I. [1.315–3.314], *P* = 0.047) were independent risk factors for DFS. Both log-rank test and Cox proportional hazards model showed that expression of SKA3 was significantly correlated with the prognosis of breast cancer. These results are described in Table 2 and Fig. 4.

**Table 2:** Univariate and multivariate analyses of disease-free survival in breast cancer.

Parameters	Univariate analysis			Multivariate analysis		
	HR	95% C.I.	*P*-value	HR	95% C.I.	*P*-value
SKA3	1.514	[1.105–2.074]	**0.010**	1.410	[1.018–1.953]	**0.039**
Age	0.974	[0.947–1.002]	0.070	0.988	[0.954–1.009]	0.176
T classification	2.088	[1.315–3.314]	**0.002**	1.984	[1.010–3.893]	**0.047**
N classification	1.496	[1.034–2.166]	**0.033**	1.350	[0.765–2.386]	0.301
TNM stage	2.048	[1.200–3.495]	**0.009**	0.920	[0.318–2.642]	0.872
Molecular subtype	1.502	[0.761–2.966]	0.241	1.269	[0.633–2.541]	0.502

**Note:**

Statistically significant *P* values are given in bold, *P* < 0.05; HR, hazard ratio; C.I., confidence interval.

### Identification of SKA3-related signaling pathways by GSEA

**Figure 5:**
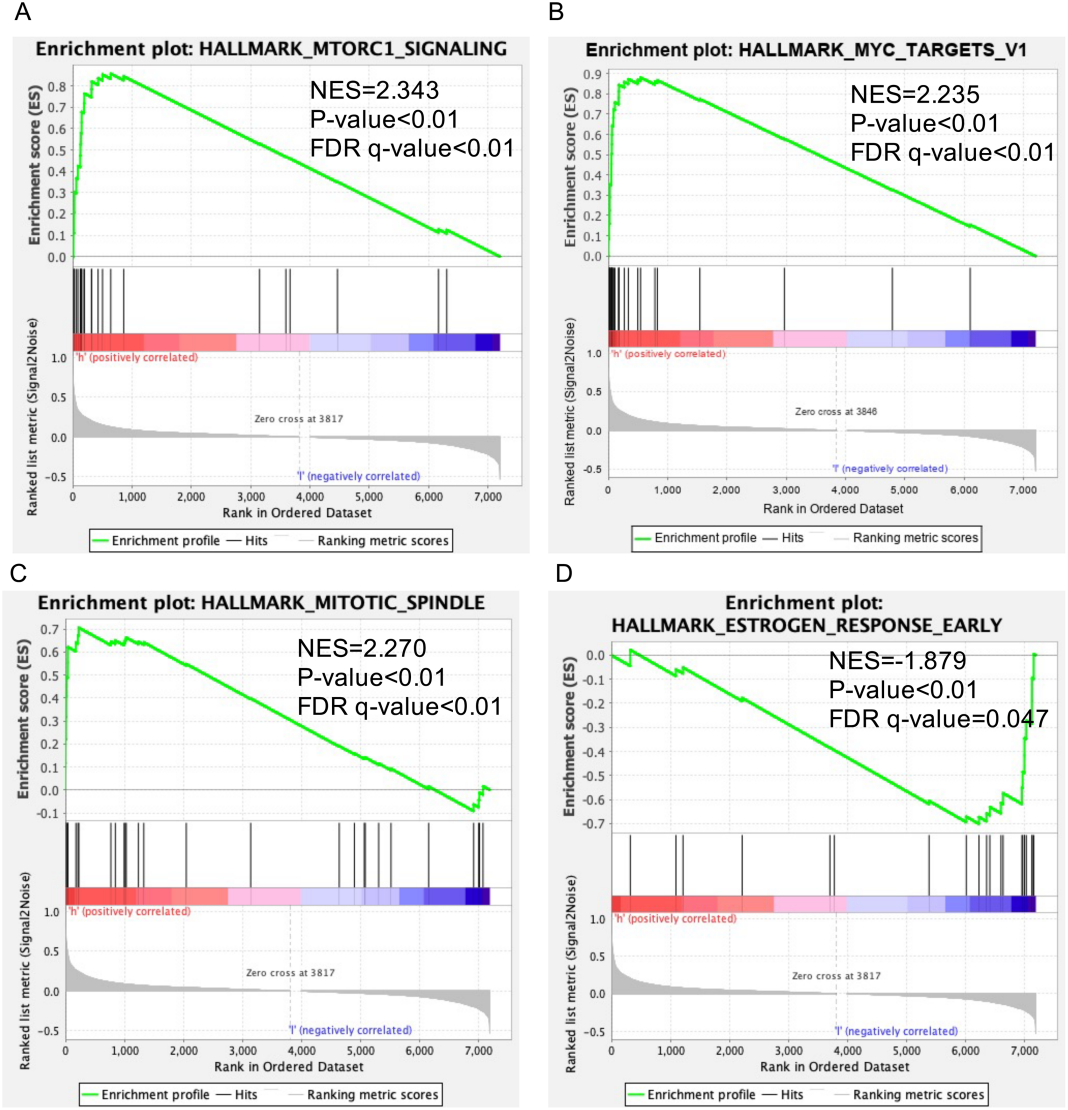
Enrichment plots of GSEA in breast cancer with a high SKA3 expression phenotype. GSEA results showed that (A) mTORC1 signaling pathway, (B) MYC targets v1, (C) mitotic spindle, (D) estrogen response early.

Data sets from GSEA showed significant differences (|NES| > 1, FDR < 0.25, NOM *P* < 0.05) in MSigDB Collection. The significant pathways by GSEA included mTORC1 signaling pathway (Fig. 5A), MYC targets v1 (Fig. 5B), mitotic spindle (Fig. 5C), estrogen response early (Fig. 5D).

### The verification of SKA3 by RT-PCR and ELISA

**Figure 6:**
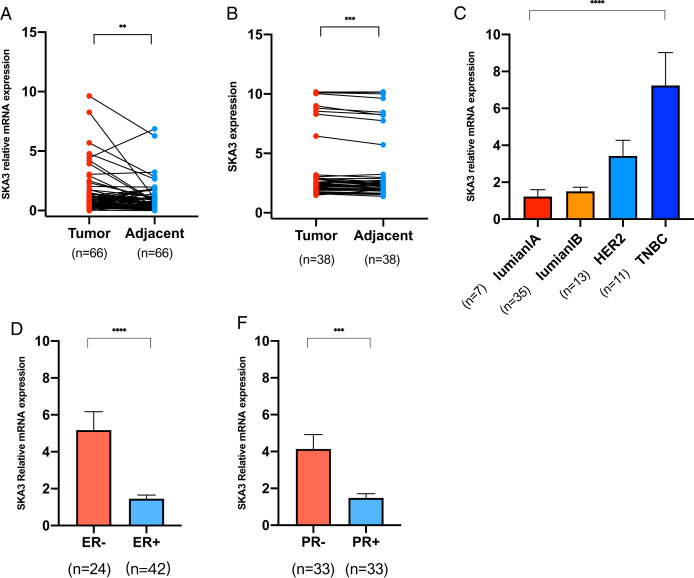
RT-qPCR and ELISA analysis. (A) RT-qPCR analysis of SKA3 mRNA expression in 66 pairs of breast cancer tissues and paired adjacent tissues. (B) ELISA analysis of SKA3 expression in 38 pairs of BC tissues and paired adjacent tissues. (C) RT-qPCR analysis of SKA3 mRNA in molecular subtype. (D) RT-qPCR analysis of SKA3 mRNA in ER status. (E) RT-qPCR analysis of SKA3 mRNA in PR status. Note: **P < 0.01; ***P < 0.001; ****P < 0.0001.

The RT-qPCR showed that the SKA3 mRNA in 66 pairs of breast cancer tissues was higher than that in adjacent tissues (*P* < 0.01, Fig. 6A). Besides, ELISA showed that SKA3 protein expression in 38 pairs of BC tissues was over-expressed in breast cancer tissues than that in adjacent tissues, (*P* < 0.001, Fig. 6B), which is corresponding to RT-qPCR results. For ER and PR status, SKA3 mRNA expression is higher in ER, PR negative than ER, PR positive (*P* < 0.0001, Fig. 6C and *P* < 0.01, Fig. 6D). In all molecular subtypes, SKA3 mRNA expression is the highest in TNBC breast cancer and the lowest in luminalA breast cancer (*P* < 0.0001, Fig. 6F). And no difference was found in SKA3 expression in age, T classification, N classification, TNM stage, and HER2 status by statistical significance of our results (Fig. S1). As can be seen in Table 3, SKA3 mRNA expression was significantly correlated with ER status, PR status, and molecular subtype in BC patients.

**Table 3:** Relationship between SKA3 mRNA expression by RT-qPCR and clinicopathologic parameters.

Parameters	SKA3-high (*n* = 33)	SKA3-low (*n* = 33)	χ^2^	*P*-value
Age				
≤40	7 (21.2%)	4 (12.1%)	0.982	0.322
>40	26 (78.8%)	29 (87.9%)		
T classification^Δ^				
T1	12 (36.4%)	13 (39.4%)	0.596	1.397
T2	17 (51.5%)	18 (54.5%)		
T3	4 (12.1%)	2 (6.1%)		
N classification^Δ^				
N0	24 (72.7%)	21 (63.6%)	1.520	0.853
N1	6 (18.2%)	8 (24.2%)		
N2	3 (9.1%)	3 (9.1%)		
N3	0 (0%)	1 (3.0%)		
ER				
Positive	14 (42.4%)	28 (84.8%)	12.833	**<0.001**
Negative	19 (57.6%)	5 (15.2%)		
PR				
Positive	11 (33.3%)	22 (66.7%)	7.333	**0.007**
Negative	22 (66.7%)	11 (33.3%)		
HER2				
Positive	12 (36.4%)	7 (21.2%)	1.848	0.174
Negative	21 (63.6%)	26 (78.8%)		
Histological type				
Non-invasive ductal carcinoma	6 (18.2%)	6 (18.2%)	0	1
Invasive ductal carcinoma	27 (18.2%)	27 (81.8%)		
TNM stage^Δ^				
I	10 (30.3%)	10 (30.3%)	0.827	0.642
II	18 (54.5%)	20 (60.6%)		
III	5 (15.2%)	3 (9.1%)		
Ki67				
Low	5 (15.2%)	8 (24.2%)	0.862	0.353
High	28 (84.8%)	25 (75.8%)		
Menopausal status				
No	25 (75.8%)	25 (75.8%)	0	1
Yes	8 (24.2%)	8 (24.2%)		
Histological grade^Δ^				
I	1 (3.0%)	2 (6.1%)	5.763	0.104
II	13 (39.4%)	21 (63.6%)		
III	11 (33.3%)	4 (12.1%)		
Unknown	8 (24.2%)	6 (18.2%)		
Molecular subtype^Δ^				
LuminalA	3 (9.1%)	4 (12.1%)	14.468	**0.002**
LuminalB	11 (33.3%)	24 (72.7%)		
HER2 positive	9 (27.3%)	4 (12.1%)		
Triple negative	10 (30.3%)	1 (3.0%)		

**Notes:**

Δ Means Fisher test.

Bold values indicate statistically significant, *P* < 0.05.

### Differential SKA3 expressions in the TCGA Asian-specific group

**Figure 7:**
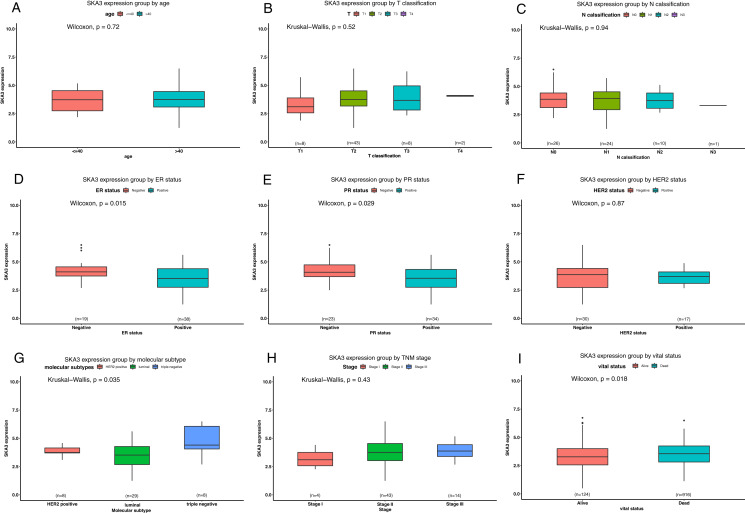
Differential SKA3 expressions in the TCGA Asian-specific group. The expression of SKA3 is grouped by age (A), T classification (B), N classification (C), ER status (D), PR status (E), HER2 status (F), molecular subtype (G), TNM stage (H), and vital status (I).

To further explore the Asian-specific group in the TCGA data, the Wilcoxon signed-rank sum test and Kruskal–Wallis test were used to access the differential expression of SKA3 in the subgroup. The results had been shown in Fig. 7. SKA3 was overexpressed in ER-negative, PR-negative, triple-negative breast cancer patients and decreased status.

### SKA3-associated PPI network

**Figure 8:**
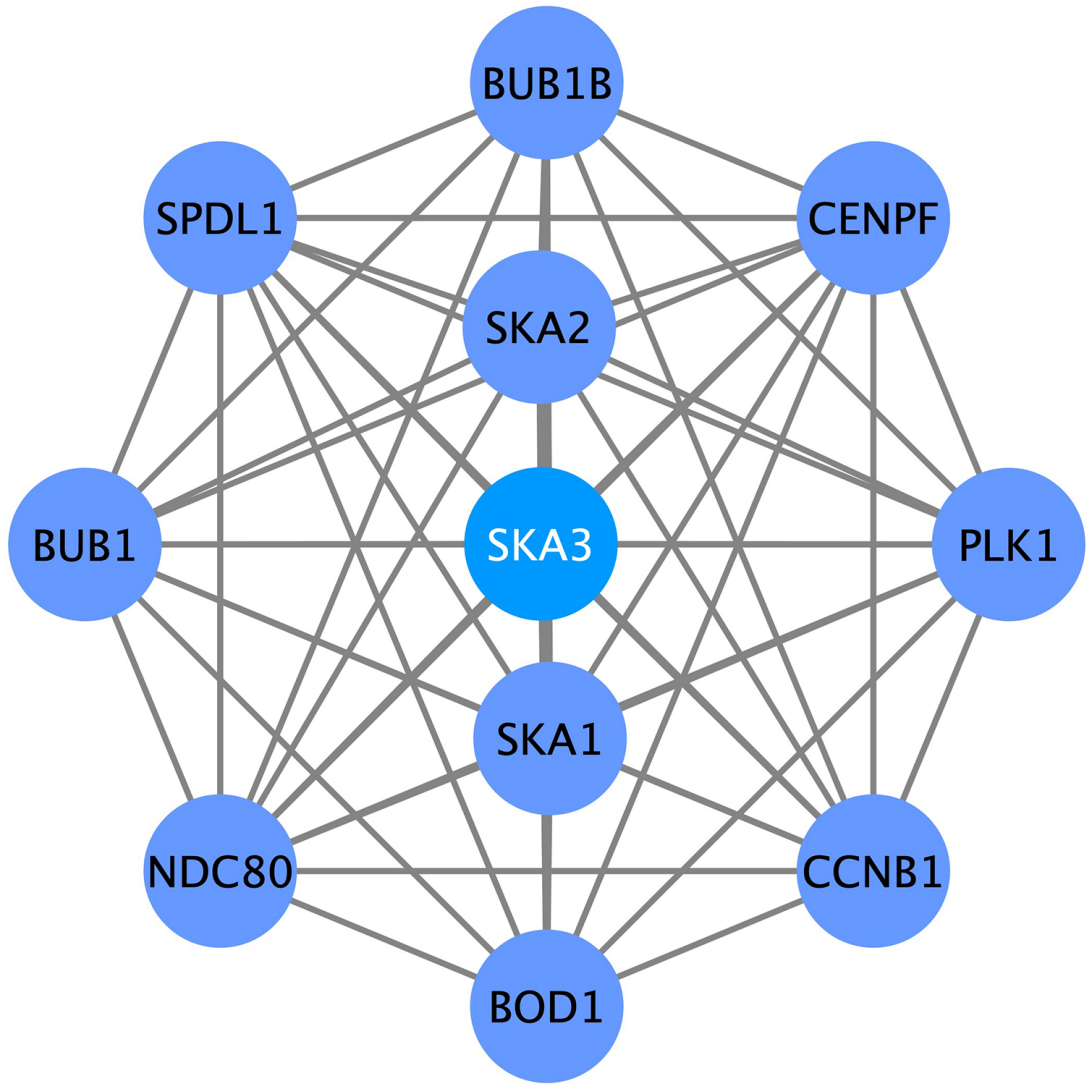
SKA3-associated PPI network. SKA1, SKA2, PLK1, CCNB1, CENPF, BUB1B, SPDL1, BUB1, NDC80 and BOD1 were related with SKA3.

To further search for the SKA3-associated PPI network in breast cancer, we performed a correlation analysis using the STRING database. Several genes had a close association with SKA3, such as SKA1, SKA2, PLK1, CCNB1, CENPF, BUB1B, SPDL1, BUB1, NDC80, and BOD1 (Fig. 8).

### High-expressed SKA3 correlates with reduced immune infiltration in breast cancer

**Figure 9:**
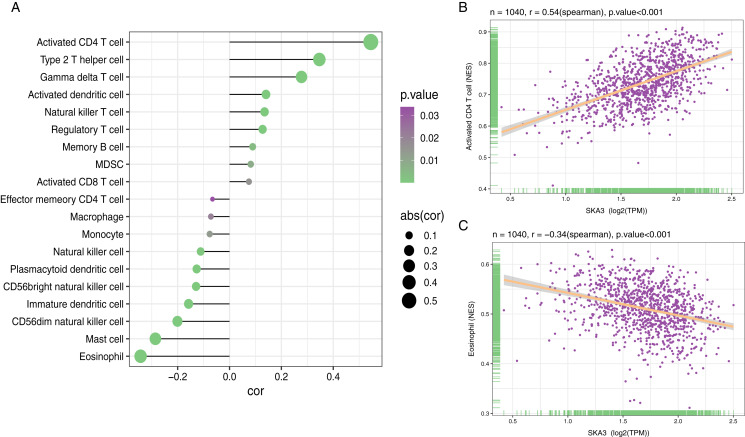
Association analysis of SKA3 gene expression and immune infiltration. (A) association analysis between SKA3 expression and immune cells; (B) association analysis of SKA3 expression with immune infiltration levels of activated CD4 T cells, and (C) eosinophils.

Among 28 gene sets, ssGSEA showed that the SKA3 expression level was positively correlated with activated CD4 T cells (r = 0.54, *P* < 0.001), while it was negatively correlated with the infiltrating level of eosinophils (r = −0.34, *P* < 0.001) (Fig. 9).

## Discussion

Many studies have confirmed that SKA3 was upregulated in numerous cancers and act as an oncogene in cervical and pancreatic cancer. However, SKA3 expression and its role in early breast cancer have not yet been reported. In our study, the Oncomine and TCGA database were used to analyze the expression of SKA3 in tumor tissues and normal tissues. The results showed that SKA3 in breast cancer tumor tissue was higher than that in normal tissues. Then, RT-qPCR and ELISA methods were used to verify the high expression of SKA3 in BC, which was corresponding with the bioinformatics findings. Besides, the findings of the present study have also coincided with the previous studies in other tumors. SKA3 was highly expressed in a variety of tumors, such as liver (Hou et al., 2019), lung (Dan-Dan et al., 2020), cervical (Hu et al., 2018), rectal cancer (Chuang et al., 2016), etc. These results suggested that SKA3 might play an important role in the formation and proliferation of tumors, and could be an oncogene in the occurrence and development of breast cancer. ER and PR status were negatively associated with SKA3 mRNA expression. In all molecular subtypes, SKA3 was the highest expression in triple-negative breast cancer and the lowest expression in luminalA, which was consistent with the previous analysis of the TCGA database. As is known to all, ER, PR positive and luminalA in breast cancer are characterized by low invasiveness, better differentiation, and slow disease progression (Han & Kang, 2010), related to the satisfactory treatment response and good clinical outcomes. However, triple-negative breast cancer is associated with a poor prognosis and is prone to lung metastasis (Turner & Reis-Filho, 2006). It was suggested that breast cancer with overexpression of SKA3 might be more malignant and aggressive.

Secondly, we sought the relationship between SKA3 and clinicopathological characteristics. It was found that SKA3 was significantly correlated with age, T classification, TNM stage, ER, PR, race, and molecular subtype based on previous public database results, which were further validated by analyzing SKA3 expression data as a continuous variable (Table 1 and Fig. 2). Although our findings showed that SKA3 correlated with ER, PR status, and molecular subtype (Fig. 6 and Table 3), they shared similarities and differences. Two reasons were taken into consideration: sample size and ethnic differences. On the one hand, the sample size included in this study was too small with only 66 cases, while the public database sample size was larger than 1,000 cases, an insufficient sample size might not demonstrate the difference. On the other hand, the dominating race of the patients in the TCGA cohort was caucasian, while in our validating cohort the dominating race was xanthoderm. As shown in our bioinformatics analysis, SKA3 was overexpressed in Asians, which might be racial differences in this gene. Thus, we explore the Asian-specific group further in the TCGA data, the results showed that SKA3 was overexpressed in ER-negative, PR-negative, triple-negative breast cancer patients and decreased status, which was roughly corresponding with our results (Figs. 6C–6F). In summary, SKA3 was overexpressed in ER-negative, PR-negative, triple-negative breast cancer both in the whole TCGA cohort, Asian-specific group in TCGA and our Asian-specific validating cohort in female breast cancer.

Kaplan–Meier survival analysis illustrated that the high-SKA3 expression group had worse overall survival, disease-free survival than the low-SKA3 expression group (Fig. 3). Also, in other tumors, it had been demonstrated that the high expression of SKA3 was closely related to the pathological and clinical features and poor prognosis of liver cancer, lung adenocarcinoma, rectal cancer, and other tumors, which was consistent with our results. Interestingly, the overexpression of SKA3 indicated a poor prognosis and an oncogene for a variety of tumor recurrence and metastasis. It was surmised that the expression level of SKA3 might serve as a candidate biomarker to evaluate the prognosis of breast cancer patients.

The potential biological functions of SKA3 in breast cancer had not yet been fully clarified. In our study, the enrichment analysis showed that the high SKA3 expression group was positively correlated with the following terms: mTORC1 signaling pathway, MYC targets v1, and mitotic spindle. Besides, the high SKA3 expression group was negatively correlated with estrogen response early which was closely related to the progression of breast cancer. mTOR complexes are mTORC1 and mTORC2, among which mTORC1 has been studied more thoroughly. mTORC1 is a downstream target of the PI3K-Akt signaling pathway. PI3K-Akt-mTORC1 signaling pathway plays an important role in the development of breast cancer and is related to cell transformation, tumorigenesis, cancer progression, and drug resistance. Previous studies reported that SKA3 promotes the proliferation and migration of lung adenocarcinoma and cervical cancer cells through the PI3K-Akt axis (Dan-Dan et al., 2020; Hu et al., 2018), suggesting that the PI3K-Akt pathway may be critical for SKA3 to promote tumor formation, invasion, and metastasis. In combination with the previous study and our GSEA results, it was speculated that SKA3 might activate the PI3K-Akt-mTORC1 pathway and promote the occurrence and development of breast cancer. It has been confirmed that MYC, as an oncogenic transcription factor, contributes to tumor cell metabolism and increases TNBC (Lawson et al., 2015; Sodir et al., 2020; Wahlstrom & Henriksson, 2015). Targeting the MYC gene provides a new research direction for the treatment of triple-negative breast cancer (Camarda et al., 2016). Schulze’s study showed that high MYC Targets Scores indicated poor survival in ER-positive and metastatic breast cancer (Schulze et al., 2020). It was speculated that SKA3 might be an upstream gene of MYC, and the high expression of SKA3 might activate MYC oncogene and promote tumor progression, which was associated with poor survival outcomes of breast cancer. The mitotic spindle is the process by which chromosomes can be accurately segregated during mitosis. SKA3 is a component of the spindle-mitochondria-related complex, which is necessary for normal chromosome separation and cell division, and plays an important role in the accurate timing of mitotic spindle migration and anaphase mitosis (Zhang et al., 2012). Anti-tumor drugs, chemotherapy drugs such as paclitaxel and vinorelbine, inhibit the growth of breast cancer by destroying the structure of mitotic spindles (Honore et al., 2003). SKA3 is a component of the spindle-mitochondria related complex, necessary for normal chromosome separation and cell division, facilitating the meiosis spindle migration and the accurate timing of anaphase mitosis. Deletion of SKA3 can lead to mitosis failure (Raaijmakers et al., 2009). In our study, the mitotic spindle was enriched in a high SKA3 expression group. It was speculated that overexpression of SKA3 might cause abnormalities of the mitotic spindle or close the mitotic checkpoint, promoting cell proliferation, leading to the generation of breast cancer. Early estrogen response is closely related to ER and PR estrogen receptor positivity and often predicts a good survival outcome. Oshi et al. (Oshi et al., 2020) showed that a high score early estrogen response was significantly associated with a better response to endocrine therapy and survival in both primary ER-positive and metastatic breast cancer. Interestingly, SKA3 overexpression predicts a poor prognosis. Considering that high expression of SKA3 was negatively correlated with “early estrogen response” in the GSEA, it strongly further suggested that SKA3 overexpression might contribute to tumor progression by inhibiting estrogen response early in BC.

SKA3-associated PPI network showed that SKA3 was related to SKA1 and SKA2. The members of the SKA family include SKA1, SKA2, and SKA3, and the heterodimer composed of the three constitute the spindle and centromere-related protein complex, which play an irreplaceable role in the stable binding of centromere and microtubules (Hanisch, Sillje & Nigg, 2006). Also, the SKA family is contributed to cancer progression. Several studies have documented that SKA1 plays an important role in the growth and proliferation of a variety of cancers, such as hepatocellular carcinoma (Chen et al., 2018), gastric cancer (Sun et al., 2014) and non-small cell lung cancer (Shen et al., 2016). SKA1 is a hub gene related to the pathologic stage of breast cancer (Fu et al., 2019). SKA1 and SKA3 genes are immunotherapy-related biomarkers in breast cancer and breast cancer stem cells (Wang et al., 2020). SKA2 is involved in mitosis and is essential for its regulation. Mitotic abnormalities are characteristic of most tumors (Suzuki et al., 2018). PRR11 and SKA2 gene pair are overexpressed in breast cancer and esophageal carcinoma (Chen et al., 2020; Wang et al., 2019), which accelerate proliferation, migration, and invasive capabilities. SKA2 can mediate the proliferation, migration, and invasion of breast cancer cells through EMT (Ren et al., 2019). MiR-520d-3p antitumor activity in breast cancer *via* post-transcriptional regulating SKA2 (Ren et al., 2018).

The tumor cell microenvironment plays a key role in the development of tumors. We identified the relationship between the infiltrating level of immune cells and SKA3 by ssGSEA. Interestingly, SKA3 expression was shown to be significantly associated with the infiltrating level of activated CD4 T cells and eosinophils. It seemed that SKA3 expression affected immune cell content in the breast cancer tumor microenvironment, exerting both antitumor and protumor functions. Activated immune cells in the tumor microenvironment secrete pro-inflammatory cytokines and chemokines could promote tumor cell proliferation (Ferrari et al., 2019). In addition, the specific cellular immune response provoked by CD4 + T cells causes chronic inflammation in tissues. Tumor-infiltrating lymphocytes can secrete proinflammatory cytokines and promote angiogenesis, leading to breast cancer metastasis as result (Esquivel-Velazquez et al., 2015). Also, a study showed that TNBCs had a higher number of CD4 + T cells than non- non-TNBCs (Kim et al., 2013). TNBC is associated with a higher risk of aggression and distant metastasis (Turner & Reis-Filho, 2006). Eosinophil infiltration is considered a favorable prognosis in breast cancer (Sakkal et al., 2016). A previous study indicated that low baseline eosinophil count was related to a higher recurrence rate in 419 patients diagnosed with breast cancer (Ownby et al., 1983). Similar research showed that relative eosinophil count (REC) was associated with a worse prognosis in 930 breast cancer patients (Onesti et al., 2020). Moreover, there was a positive correlation between REC and pathological complete remission and survival rate in TNBC and hormone receptor-negative/HER2-positive breast cancer patients (Onesti et al., 2018). We speculated that high-expressed SKA3 might impact the eosinophil and activated CD4 T cells, triggering a disadvantageous immune response, leading to a poor prognosis in breast cancer.

On top of that, SKA3 expression may be an independent predictor of a poor disease survival prognosis in breast cancer patients. However, this study had several limitations. The sample size included in the experimental validation part was relatively small and required a larger sample size. The collected breast cancer tissues were fresh samples and the follow-up time was short. Further studies are needed to explore the mechanism of SKA3 in breast cancer.

## Conclusions

In summary, SKA3 is overexpressed in breast cancer. High SKA3 expression correlates with poor prognosis and immune infiltrates in breast cancer and may become a biomarker for the prognosis of breast cancer. The findings may help us obtain deeper insights into therapeutic targeting for breast cancer.

## Supplemental Information

10.7717/peerj.12506/supp-1 Supplemental Information 1 Differential SKA3 expressions in the TCGA Asian-specific group. The expression of SKA3 is grouped by (A) age, (B) T classification, (C) N classification, (D) TNM stage, (F) HER2 status.

10.7717/peerj.12506/supp-2 Supplemental Information 2 Code.

10.7717/peerj.12506/supp-3 Supplemental Information 3 Raw data.

10.7717/peerj.12506/supp-4 Supplemental Information 4 DNA Sequencing of SKA3.

## Abbreviation list

SKA1: Spindle and kinetochore associated complex subunit 1
SKA2: Spindle and kinetochore associated complex subunit 2
SKA3: Spindle and kinetochore associated complex subunit 3
TCGA: The Cancer Genome Atlas
RT-qPCR: quantitative reverse transcription PCR
ELISA: enzyme-linked immunosorbent assay
ER: estrogen receptor
PR: progesterone receptor
TNM: tumor-node-metastasis
T: tumor
N: node
M: metastasis
HER-2: human epidermal growth factor receptor-2
BC: breast cancer
TNBC: triple-negative breast cancer
REC: relative eosinophil count
Ki-67: proliferation-related antigen Ki-67
ES: enrichment score
NES: normalized enrichment score
NOM: nominal
FDR: false discovery rate

## Additional Information and Declarations

### DNA Deposition

The following information was supplied regarding the deposition of DNA sequences: The homo sapiens actin beta sequence is available at GenBank: NM_001101.3. The SKA3 primer pair is available at PrimerBank: 260763909c1. The Primer set ID HA067803 is available at NCBI’s Primer-BLAST, using “TGGCACCCAGCACAATGAA” for the forward sequence and “CTAAGTCATAGTCCGCCTAGAAGCA” for the reverse primer sequence.

### Data Availability

The following information was supplied regarding data availability: The raw measurements are available in the Supplemental Files.
