# Supplementary figures and images for: Overexpression of SKA3 correlates with poor prognosis in female early breast cancer
Source: PeerJ. 2021 Dec 13;9:e12506. doi: 10.7717/peerj.12506 (PMC8675262; doi:10.7717/peerj.12506)

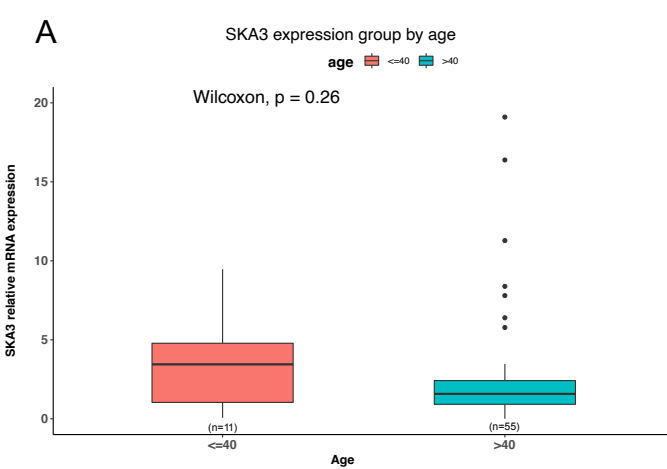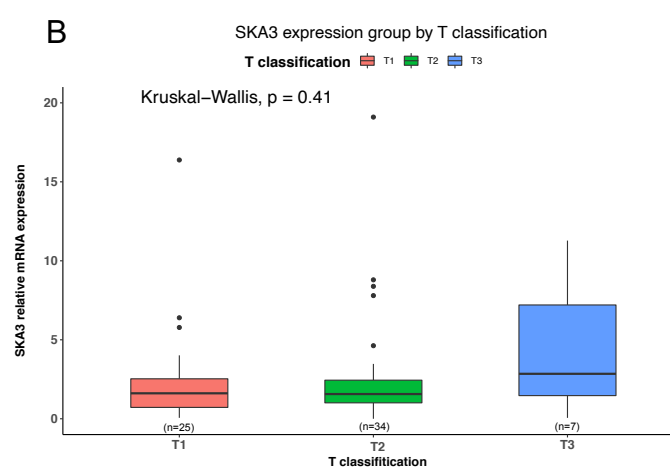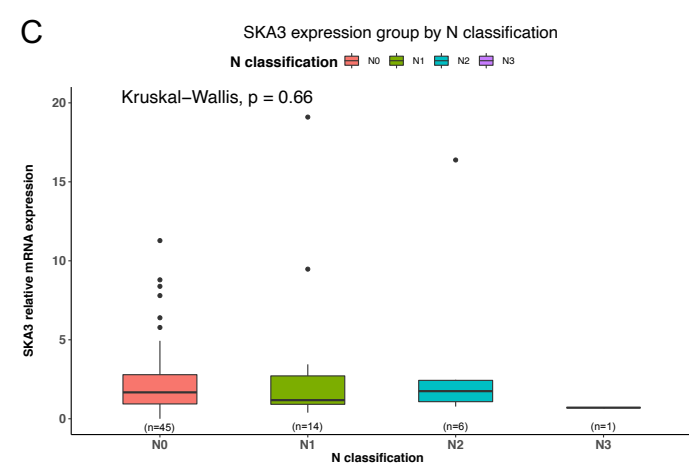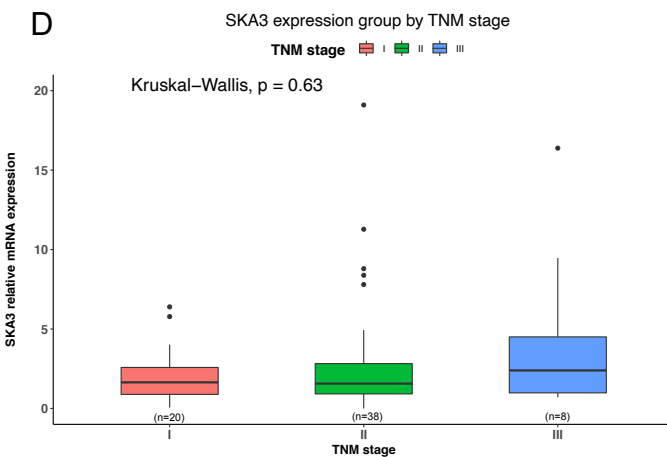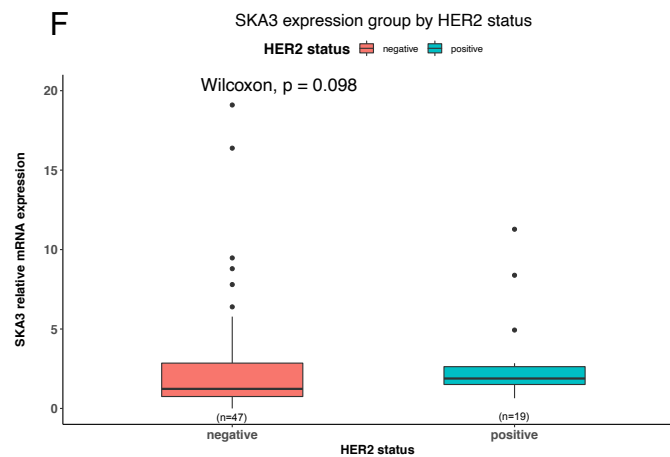

Supplement: Supplemental Information 1 — The expression of SKA3 is grouped by (A) age, (B) T classification, (C) N classification, (D) TNM stage, (F) HER2 status. [file peerj-09-12506-s001.pdf]
