# Supplementary material for: Overexpression of SKA3 correlates with poor prognosis in female early breast cancer
Source: PeerJ. 2021 Dec 13;9:e12506. doi: 10.7717/peerj.12506 (PMC8675262; doi:10.7717/peerj.12506)
Supplement: Supplemental Information 4 [file peerj-09-12506-s004.html]

PrimerBank Search Result


|  |  |  |  |  |  |  |  |  |  |  |
| --- | --- | --- | --- | --- | --- | --- | --- | --- | --- | --- |
|  | | | | | | | | | | |
|  | |  | | --- | |  |  |  |  |  |  |  |  |  | | --- | --- | --- | --- | --- | --- | --- | | PrimerBank | | | | | | |    **The following primer pair is found for 260763909c1**    |  |  | | --- | --- | | **Gene Descriptions:** | | | **NCBI GeneID** | 221150 | | **GenBank Accession** | NM\_145061 | | **NCBI Protein Accession** | NP\_659498 | | **Species** | Human | | **Coding DNA Length** | 1239 | | **Gene Description** | Homo sapiens spindle and kinetochore associated complex subunit 3 (SKA3), transcript variant 1, mRNA. |     |  |  |  |  |  | | --- | --- | --- | --- | --- | | **Primer Pair Descriptions:** | | | | | | **PrimerBank ID** | 260763909c1 | | **Amplicon Size** | 176 | |  | **Sequence** (5' -> 3') | **Length** | **Tm** | **Location** | | **Forward Primer** | TACACGAGCAAGAAGCCATTAAC | 23 | 60.9 | 332-354 | | **Reverse Primer** | GGATACGATGTACCGCTCAAGT | 22 | 61.6 | 507-486 | | **Location in Coding Sequence** (primers and amplicon highlighted) | | | | | | ```     1 atggacccta tccggagctt ctgcgggaag ctgcggtctc tggccagcac gctggactgc    61 gagacggccc ggctgcagcg agcgctggac ggagaggaaa gcgactttga agattatcca   121 atgagaattt tatatgacct tcattcagaa gttcagactc taaaggatga tgttaatatt   181 cttcttgata aagcaagatt ggaaaatcaa gaaggcattg atttcataaa ggcaacaaaa   241 gtactaatgg aaaaaaattc aatggatatt atgaaaataa gagagtattt ccagaagtat   301 ggatatagtc cacgtgtcaa gaaaaattca gtacacgagc aagaagccat taactctgac   361 ccagagttgt ctaattgtga aaattttcag aagactgatg tgaaagatga tctgtctgat   421 cctcctgttg caagcagttg tatttctgag aagtctccac gtagtccaca actttcagat   481 tttggacttg agcggtacat cgtatcccaa gttctaccaa accctccaca ggcagtgaac   541 aactataagg aagagcccgt aattgtaacc ccacctacca aacaatcact agtaaaagta   601 ctaaaaactc caaaatgtgc actaaaaatg gatgattttg agtgtgtaac tcctaaatta   661 gaacactttg gtatctctga atatactatg tgtttaaatg aagattacac aatgggactt   721 aaaaatgcga ggaataataa aagtgaggag gccatagata cagaatccag gctcaatgat   781 aatgtttttg ccactcccag ccccatcatc cagcagttgg aaaaaagtga tgccgaatat   841 accaactctc ctttggtacc tacattctgt actcctggtt tgaaaattcc atctacaaag   901 aacagcatag ctttggtatc cacaaattac ccattatcaa aaacaaatag ttcatcaaat   961 gatttggaag ttgaagatcg tacttcgttg gttttaaatt cagacacatg ctttgagaat  1021 ttaacagatc cctcttcacc tacgatttct tcttatgaga atctgctcag aacacctaca  1081 cctccggaag taactaaaat tccagaagat attctccagc ttttatcaaa atacaactca  1141 aacctagcta ctccaatagc aattaaagca gtgccaccca gtaaaaggtt ccttaaacat  1201 ggacagaaca tccgagatgt cagcaacaaa gaaaactga ``` | | | | |      --- | | | | | | | | |
